# Practical Hydration Solutions for Sports

**DOI:** 10.3390/nu11071550

**Published:** 2019-07-09

**Authors:** Luke N. Belval, Yuri Hosokawa, Douglas J. Casa, William M. Adams, Lawrence E. Armstrong, Lindsay B. Baker, Louise Burke, Samuel Cheuvront, George Chiampas, José González-Alonso, Robert A. Huggins, Stavros A. Kavouras, Elaine C. Lee, Brendon P. McDermott, Kevin Miller, Zachary Schlader, Stacy Sims, Rebecca L. Stearns, Chris Troyanos, Jonathan Wingo

**Affiliations:** 1 Korey Stringer Institute, Department of Kinesiology, University of Connecticut, Storrs, CT 06269, USA; 2 Faculty of Sport Sciences, Waseda University, Saitama 359-1192, Japan; 3 Department of Kinesiology, University of North Carolina at Greensboro, Greensboro, NC 27402, USA; 4 Department of Kinesiology, University of Connecticut, Storrs, CT 06269, USA; 5 Gatorade Sports Science Institute, Barrington, IL 60010, USA; 6 Sports Nutrition, Australian Institute of Sport, Canberra, ACT 2617, Australia; 7 Sports Science Synergy, LLC, Franklin, MA 02038, USA; 8 U.S. Soccer, Chicago, IL 60616, USA; 9 Centre for Human Performance, Exercise and Rehabilitation, Brunel University London, Uxbridge UB8 3PH, UK; 10 Hydration Science Lab, College of Health Solutions, Arizona State University, Phoenix, AZ 85004, USA; 11 Department of Health, Human Performance and Recreation, University of Arkansas, Fayetteville, AR 72701, USA; 12 Department of Rehabilitation and Medical Sciences, Central Michigan University, Mount Pleasant, MI 48859, USA; 13 Department of Exercise and Nutrition Sciences, University at Buffalo, Buffalo, NY 14214, USA; 14 Faculty of Health, Sport and Human Performance, University of Waikato, Hamilton 3216, New Zealand; 15 International Institute of Race Medicine, Plymouth, MA 02360, USA; 16 Department of Kinesiology, University of Alabama, Tuscaloosa, AL 35487, USA

**Keywords:** fluid replacement, athletics, exercise

## Abstract

Personalized hydration strategies play a key role in optimizing the performance and safety of athletes during sporting activities. Clinicians should be aware of the many physiological, behavioral, logistical and psychological issues that determine both the athlete’s fluid needs during sport and his/her opportunity to address them; these are often specific to the environment, the event and the individual athlete. In this paper we address the major considerations for assessing hydration status in athletes and practical solutions to overcome obstacles of a given sport. Based on these solutions, practitioners can better advise athletes to develop practices that optimize hydration for their sports.

## 1. Introduction

Maintaining euhydration, the state of preserving body water within its optimal homeostatic range, is essential to sustain life. Water contributes 50–70% of total body mass and is compartmentalized within both intracellular (65%) and extracellular (35%) spaces [1]. Euhydration is typically maintained over the course of day-to-day life via behavioral and biological controls [2]. However, exercise can cause an acute disruption to fluid balance, challenging the athlete’s goal of optimal performance and safety during exercise, especially in hot environmental conditions. The process of incurring a fluid deficit is known as dehydration, while the outcome is defined as hypohydration. The loss of body water during exercise exacerbates physiological and perceptual strain [3,4,5,6,7,8,9,10] and it is well established that these changes can impair endurance performance, particularly in hot environments and may increase the risk of exertional heat illness [11,12,13,14,15,16,17].

While the sensation of thirst, a centrally mediated response to body water deficits, is useful in dictating the need for fluid intake during daily life, thirst is relatively insensitive in acutely tracking hydration status during exercise [18,19]. Maintaining an optimal state of hydration during exercise becomes more complicated depending on the sport, type of activity and availability of fluid. Optimal hydration is dependent on many factors but can generally be defined during exercise as avoiding losses greater than 2–3% of body mass while also avoiding overhydration [15]. Furthermore, during exercise, it is not uncommon for individuals to involuntarily dehydrate, in which they consume less fluid than their fluid needs. Excessive fluid intake can also be problematic, with hyponatremia developing in severe cases of overhydration [15]. Inappropriate management of fluid intake resulting in hypohydration, or hyperhydration, can be detrimental for performance and in some circumstances, increases health risk.

**Figure 1:**
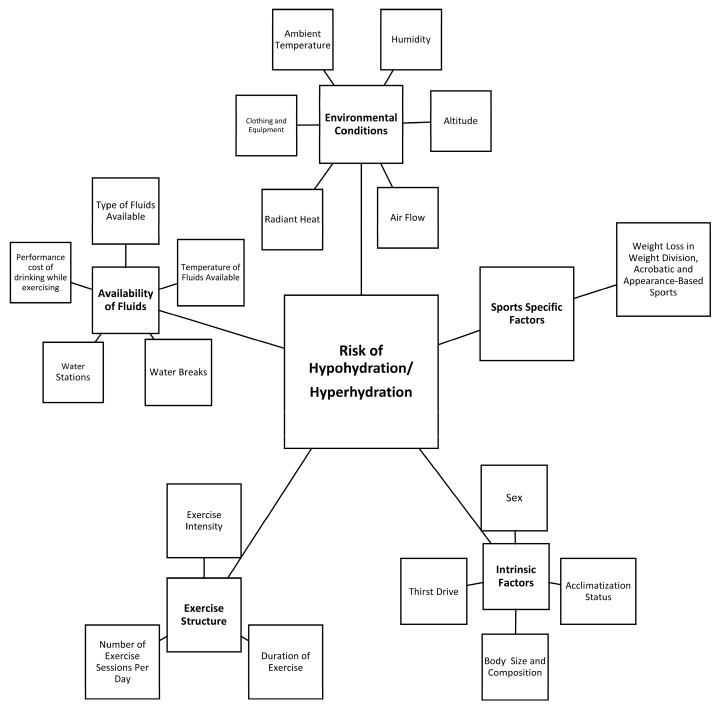
Factors that contribute to the risk of hypohydration or hyperhydration during exercise.

Current consensus recommends that good hydration practices include: (1) beginning exercise in a state of euhydration, (2) preventing excessive hypohydration during exercise, and (3) replacing remaining losses following exercise prior to the next exercise bout [15,20,21]. These practices attenuate the adverse effects of acute dehydration on physical activity and health [15]. However, it is acknowledged that fluid needs are individualistic and rely on factors such as personal sweat rate, exercise mode, exercise intensity, environmental conditions and exercise duration (Figure 1) [14,15,22,23,24,25]. Furthermore, characteristics and rules unique to each sport environment in which it is played, event uniform and equipment, and the availability of fluid during both training and competition may greatly influence the ability to optimize hydration during activity. The Korey Stringer Institute and Gatorade convened a meeting to address these issues as they relate to athletes. The purpose of these proceedings is to discuss practical strategies to assess and tailor hydration for sports. This manuscript will specifically focus on the factors that underlie fluid needs and provide guidance to clinicians and practitioners on how to plan for these needs in the context of a given activity.

## 2. Hydration Assessment

Hydration assessment can be utilized to indicate one’s current hydration state, but if taken serially, it can also be used to track changes in hydration and indicate fluid needs (i.e., during a bout of physical activity). While various methods of hydration assessment exist, there is no single method that can serve as a criterion measure to assess hydration status in all settings (i.e., day-to-day life and exercise, etc.). Plasma osmolality, changes in plasma volume, and the volume, osmolality and specific gravity of urine are the most commonly published metrics to assess changes in hydration status in clinical settings [26]. The use of these measures in field applications is often impractical, due to the methods or equipment needed to acquire the measure (e.g., a needle stick to draw blood or providing a urine sample) as well as the sensitivity of the measure.

In field applications, the careful assessment of changes in body mass over a bout of physical activity provides a reasonably accurate assessment of body water deficits incurred during the session, since sweat loss and fluid intake during the session underpin the major changes in body mass and body water content. This is true for most sporting activities conducted over a duration of <2–3 h; however, during very prolonged and strenuous exercise (e.g., ultra-endurance races), other factors that cause mass changes, metabolic water production and the liberation of stored water become numerically important and undermine the utility of this assessment [15,27,28]. A comparison of body mass pre- and post-exercise will help guide the athlete in understanding whether their hydration strategy during activity was effective in achieving acceptable fluid balance as well as knowing the volume of fluids that are needed following exercise to return to baseline hydration levels prior to the next exercise session. The methodology for assessing sweat losses is to assess the athlete’s body mass before and after exercise with care to avoid or account for substantial amounts of fluid trapped in hair and clothes. Accounting for any fluid consumed or urine excreted, the difference between the masses can be used to calculate the amount of sweat lost as well as the residual fluid deficit that should be addressed in post-exercise recovery plans [29].

A useful paradigm for tracking daily changes in hydration status in sporting situations is to consider a combination of assessments to track daily changes. The monitoring of daily changes in body mass, coupled with urine color and thirst sensation status provides adequate sensitivity for most athletic situations [30]. Cheuvront and Kenefick established useful criteria for these variables as body mass changes greater than 1.1%, a conscious desire for water (thirst), and dark-colored urine (>5 a.u. on an 8-a.u. scale [31]) indicating varying degrees of fluid inadequacy [32]. Two of these factors combined suggest daily fluid intake is likely inadequate, while all three factors indicate that daily fluid intake is very likely inadequate. It should be noted that this assessment technique is based on first morning values and requires baseline body mass values to provide the most useful information to athletes.

*Practical Solutions:*

(1) Carefully monitor acute changes in body mass over an exercise bout to determine sweat rate, adequacy of fluid replacement and fluid needs for recovery for that session. Consider how well this can be used to evaluate general hydration strategies in similar situations.
(2) Use changes in body mass, urine color and thirst upon awakening to track daily changes in hydration status.

## 3. Exercise Structure

Body fluid loss during sport or exercise largely results from sweating. Net fluid balance is modulated to a certain extent by drinking. Rate of sweating is primarily a function of metabolic heat production [33], but can be modified by environment, clothing, acclimatization and hydration status [7,34]. As the primary mechanism of heat dissipation in many environments, the evaporation of sweat is vital for regulating body temperature, even during exercise in temperate weather. However, this heat dissipation is accompanied by typical fluid losses of ~0.5–1.9 L/h [35].

Exercise intensity is the main factor that determines metabolic heat production, meaning that the rate of fluid losses from sweat for a given exercise session can be partially explained by the intensity of the exercise [36]. Total fluid losses are a result of the sweat rate of a given exercise intensity and the total duration of that activity [27]. In most circumstances there is an inverse relationship between the exercise intensity of a session and the duration of that session. However, given the wide variability in individual sweat rates, the unique interplay between intensity, duration and sweat rate must be considered in unison. For example, a runner with a 2 L/h sweat rate who completes a marathon in 2 h will accumulate the same fluid losses as a runner with a 1 L/h sweat rate that completes the race in 4 h. In Table 1 and Table 2, we define exercise intensity of a range of sporting activities into three distinct categories (High, Moderate, Low), based on typical practice and competition structures on the principles described above but comprehensive plans should consider individual athletes.

**Figure 2:**
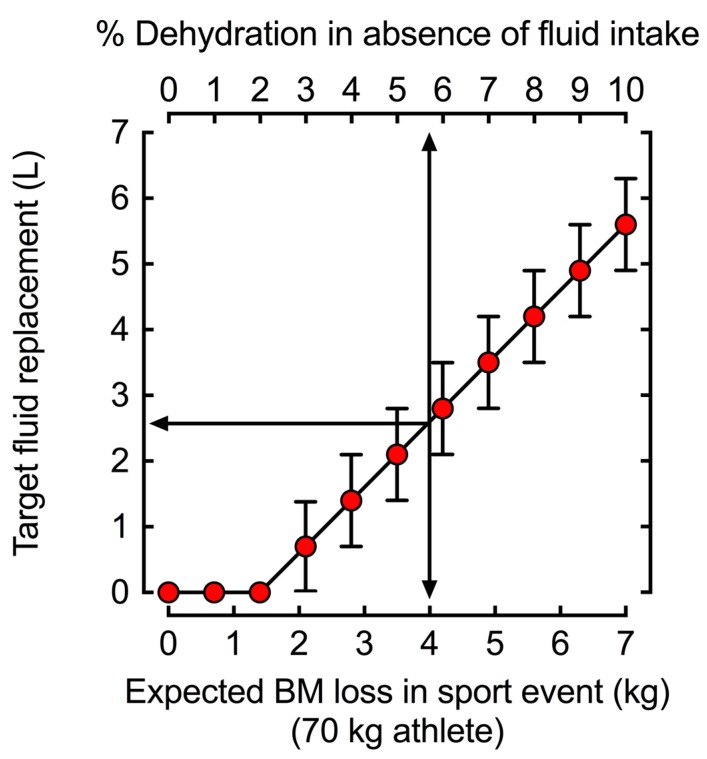
Target fluid replacement estimates to prevent >2 ± 1 % body mass (BM) loss as water (i.e., dehydration). The 70 kg athlete in the example would need to drink a volume of fluid equal to 2.6 ± 0.7 L to prevent >2 ± 1% dehydration when losing 4 L of body water, such as during a marathon (42.1 km). During shorter distances such as 5 or 10 km when fluid losses are unlikely to reach or exceed 2% dehydration, the same athlete would not need to ingest fluids during competition as fluid losses accumulate to <2% dehydration.

For sports like cycling and running, the influence of exercise intensity and duration on fluid needs is very easy to determine given the consistent nature of exercise. Figure 2 demonstrates the relationship between duration and target fluid replacement for steady-state exercise. However, as shown in Table 1, some of the most common sports involve supramaximal exercise in short bursts with longer breaks. In this case, individuals should regard an overall average of exercise intensity rather than the maximal effort during the exercise bout when determining optimal fluid balance.

It should also be noted that exercise intensity influences gastric emptying rate [37]. Individuals striving to closely match sweat losses with fluid consumption can be challenged by maximal gastric emptying rates. However, when vigorous exercise is conducted (>70% VO_2max_), gastric emptying decreases predictably, most likely based on decreased splanchnic perfusion [37].

*Practical Solutions:*

(1) Increased fluid intake is necessary with prolonged or intense exercise due to increased sweat production.
(2) During vigorous exercise (>70% VO_2max_) understand that gastric emptying may limit fluid absorption. Athletes can train their gut to improve gastrointestinal comfort or adopt strategies to increase fluid intake before and after exercise.

## 4. Environment

At a fixed exercise intensity, the ambient environment further modulates sweat rate. The magnitude of evaporative, radiant, convective and conductive heat exchange between the body and the environment is a function of the gradients between the environment and the skin which provides the main physiological interface for heat exchange. A number of factors contribute to sweat rate including ambient and radiant temperature, humidity, clothing, and air velocity [34], all of which differ depending on the sport or activity (Table 1 and Table 2). Therefore, individuals exercising in hot-humid environments with direct sunlight and minimal airflow will produce near maximal sweat rates and be at the greatest risk of hypohydration. Wet-bulb globe thermometry (WBGT) accounts for these environmental factors and can help inform fluid replacement decisions [38].

All clothing provides insulation and presents a barrier to heat loss, resulting in increased sweat rates to provide similar cooling to an unclothed situation [34]. Thus, sports/activities with specific clothing requirements, such as American football [39,40], are at greater risk of body fluid loss compared to similar activities in which clothing is minimal. Synthetic wicking materials can increase sweat fluid losses compared to cotton garments [41], potentially decreasing the thermal load but increasing the risk of dehydration. When the effects of hot, humid environmental conditions are combined with clothing and equipment, individuals can achieve near maximal sweat rates which can create a significant fluid deficit rapidly [40].

*Other Environmental Considerations:*

Exercising in the cold, or at high altitudes merits special considerations when determining the fluid needs of athletes. Athletes must also be vigilant and mindful of their fluid needs during exercise in the cold. Exercise in the cold can still produce copious sweating, especially when heavy clothing is worn, while also diminishing thirst sensitivity and reducing *ad libitum* fluid consumption, thus potentially leading to impaired fluid replacement and hypohydration [42]. If possible, athletes should know their individual fluid replacement needs, based upon sweat rate measurement, during exercise in hot and cold environments to ensure they can develop a plan for competing while optimally hydrated.

Athletes unaccustomed to exercising in higher altitudes may require additional fluids. Very high altitude (4900–7600 m) exposure tends to increase water and electrolyte losses, decrease plasma volume and total body water content [43]. In both cold air and high altitudes, respiratory water losses may increase and require additional fluid consumption due to low air water vapor pressures [44]. Therefore, athletes should acclimate to altitude over several days and maintain euhydration prior to competition to ensure optimal athletic performance.

Table 1 and Table 2 summarizes typical environmental conditions found among a range of sports into three distinct categories, and how these conditions contribute to the considerations around an individualized fluid plan. Of course, there are large regional differences in environmental conditions experienced for sports at the same time of year [45]. Local measurements utilizing WBGT allow for the greatest characterization of the environmental demands placed on athletes during exercise in the heat [46].

*Practical Solutions:*

(1) Measure local environmental conditions to determine the risk of high sweat rates resulting in large fluid losses.
(2) Increase fluid-replacement during exercise in hot and humid environments to account for increased sweat losses.
(3) Account for clothing or equipment requirements when evaluating fluid needs.
(4) Modify fluid intake when exercising in cold or altitude according to an estimation of fluid losses noting that thirst may be less reliable as a guide to dehydration under these conditions.

## 5. Fluid Availability

Fluid availability refers to the factors that dictate an athlete’s ability to replace fluid losses during activity. In many instances, the characteristics of the sport have a strong influence on the ability to drink during competition and in many scenarios prevent an athlete from “drinking to thirst” [21]. Meanwhile, training activities are often easily modified to allow for some degree of fluid replacement. In many instances, water-breaks during training can be determined on the basis of work-to-rest ratios set by environmental conditions with free access to fluid throughout the break [47]. Characteristics such as flavor and temperature affect the palatability of fluids and may increase voluntary intake when they are matched to the cultural preferences of the athletes and the prevailing conditions (e.g., cool drinks in a warm environment) [48,49].

In endurance sports which provide competitors with feed zones/water stations (e.g., running events), the number of water stations on a course and the frequency with which a competitor reaches them can influence drinking behavior. The International Association of Athletics Federations recommends that stations are placed approximately every 5 km, however, many races include more frequent stations which may influence athletes’ drinking strategies and behaviors [50]. Slower athletes with lower sweat rates who compete in such events, particularly over prolonged distances or duration, are often able to drink in volumes that exceed their true fluid losses and are at particular risk of developing hyponatremia [51]. Athletes taking part in these events should be educated on the importance of fluid balance and the prevention of hyperhydration. It should also be noted that in many events lasting up to 45 min, the risk of dehydration is low due to the limited duration across which sweat losses can accumulate. For faster and/or more competitive athletes, extra elements related to drinking while performing continuous exercise must be taken into consideration. This includes considerations around gastrointestinal comfort when fluid consumed during higher-intensity and “gut joggling” activities (e.g., high-speed running vs. the more “gliding” movements of cross-country skiing or cycling). Furthermore, the time lost in slowing down or moving out of an aerodynamic position to obtain or consume a drink must be factored into the overall race performance. This creates different factors in the cost:benefit analysis of an individual’s fluid intake plan.

The official rules and competition characteristics of “stop-start” sports such as team and racket sports create other influences, and often unique scenarios, around fluid availability. In some examples (e.g., soccer, rugby), governing rules limit the availability of fluids for athletes during competition. Soccer, for example, includes two 45-min halves (with a continuous running clock) in which fluid availability is extremely limited to players. At the other end of the spectrum are sports such as baseball, basketball and tennis with frequent rest breaks within playing time (e.g., time outs, change of ends or player rotations) during which fluids can be consumed. An athlete’s drinking strategy for a competition represents a unique instance for their particular sport based on their ability to rehydrate within the rules [52]. We support recent governing body rule changes and referee decisions to add breaks to competitions (Major League Soccer, FIFA soccer matches, US Open Tennis) to facilitate safe participation by the athletes. These changes likely augment athletic performance and safety simultaneously. Individuals should understand their sport and its fluid needs/fluid availability characteristics to prepare and practice optimal fluid plans for competition. Where rule changes, or alterations are allowed, individuals and teams should attempt to ask for these alterations (e.g., extended rest periods, additional breaks) in advance to formulate an appropriate drinking strategy. In all sports, athletes should aim to practice and fine-tune their personal drinking strategy for race/competition conditions. This will help individuals to confirm its feasibility, understand their personal responses and develop any necessary behavioral practices within the expected rules of competition.

In Table 1 and Table 2, we define fluid availability into three distinct categories, based on particular sport variations. Sports were categorized as having high fluid availability if there are multiple opportunities for fluid consumption, rather than only during breaks. Low fluid availability was used to describe those activities involving governing rules, time constraints, or an inability to carry personal fluids during competition. Accessibility to fluid consumption during competition represents a major variable to be used in preparation of an optimal fluid replacement strategy.

*Practical Solutions:*

(1) During training, ensure that there is ample access to fluids that are palatable to athletes.
(2) Investigate or understand the opportunities for fluid intake during that are specific to a sport or event, and any other practical issues that determine fluid intake.
(3) Consider the risks of hyperhydration as well as hypohydration for any sporting event or individual athlete, and prepare appropriate practice and education strategies.
(4) Develop personalized fluid intake plans that incorporate fluid availability characteristics of the sport or event. Where there is a likelihood of hypohydration, be proactive and creative in making use of existing opportunities for fluid intake within sport rules and characteristics and be prepared to request for changes when there is a likelihood of a serious mismatch between fluid losses and the opportunity to address these.
(5) Practice intended competition drinking plans ahead of time to determine their suitability and allow time for readjustment.

## 6. Intrinsic Factors

A number of intrinsic factors modulate the individual variances that are observed in fluid losses. One of the greatest considerations for an individual’s sweat rate is his or her body size. Larger individuals typically have higher sweat losses, with football linemen exhibiting some of the highest recorded sweat rates [14]. Therefore, required absolute drink volumes will be higher for these athletes. An individual’s thirst drive also dictates how much they desire to drink during exercise, but this may not match their actual fluid needs. Indeed, multiple authors report that athletes voluntarily dehydrate during exercise due to discrepancies in fluid losses and drinking behavior [53,54]. Case studies of individuals who have developed hyponatremia due to excessive drinking during exercise also note that they reported thirst as an underlying contributor to their fluid intake [55].

Heat acclimatization contributes to variations in an individual’s sweating rate responses. Individuals who are heat acclimatized exhibit greater sweat rates which can pose a greater risk of hypohydration [56]. Although the increased sweat provides extra heat dissipation, it also requires extra fluid intake.

Women may be at greater risk for exercise-induced hyponatremia. This risk has been attributed to their lower body weight and size, excess water ingestion, and longer racing times relative to men [57]. The greater incidence of hyponatremia in women is unlikely due to their greater levels of estradiol in plasma and tissue. Although female sex-hormones can also influence neural and hormonal control of thirst, fluid intake, sodium appetite and sodium regulation [58,59], there is no evidence that anything beyond stature and drinking behavior significantly impact their risk.

*Practical Solutions:*

(1) Consider body size, acclimatization status and thirst drive when developing hydration plans for individual athletes.

## 7. Sport-Specific Factors

### Weight Division, Acrobatic and Appearance-Based Sports

The culture and normal behaviors surrounding specific sports can greatly affect the hydration practices of its athletes. The three most prominent examples of the cultural effects of sports on hydration practices are weight division sports, acrobatic sports and appearance-based sports. In weight division sports (e.g., combat sports, horse racing, lightweight rowing, etc.), the practice of deliberately dehydrating to manipulate body mass to meet lighter competition weight classifications is common [60]. In many cases, athletes not only sacrifice their performance through these practices but also endanger their health and well-being. In a similar fashion, sports where body-image and appearance are emphasized (e.g., cheerleading, body building and gymnastics), dangerous practices, such as extreme fluid restriction may be used by athletes to cheat “unofficial” weight checks that are self-instigated or expected within their training environment. Finally, “acrobatic” feats such as gymnastics, jumping and climbing are aided by a high power to weight ratio, but should not rely on severe hypohydration to achieve this. Excessive use of dehydration to manage body mass goals should be corrected to avoid long-term health complications [61].

*Practical Solutions:*

(1) Promote healthy weight management strategies in weight division, acrobatic and appearance-based sports that minimize manipulation of body water.

## 8. Conclusions

Based on the factors in the above sections, along with published literature on typical fluid balance observations in various sports [62,63], we assigned risks of hypohydration to the sports in Table 1 and Table 2. These determinations can be used as a general guideline for sports that pose large risks for fluid imbalances that may limit sport performance. The factors for individual situations or geographical locations may vary and should be considered based on the principles mentioned above to tailor the necessary fluid replacement accommodations. An example of using this paradigm to develop a hydration plan can be found in Table 3.

In this paper we present a paradigm that can be used by clinicians and practitioners to develop hydration strategies for sports based on fluid availability, environment and exercise intensity. These tools are provided to inform hydration education and practices in a dynamic and individualized manner so that athletes can adapt to different circumstances and optimize performance.

## Figures and Tables

**Table 1:** Team Sport Factors That Influence Hypohydration.

Sport	Availability of Fluid		Environment		Intensity		Hypohydration Risk	
	Training	Competition	Training	Competition	Training	Competition	Training	Competition
Basketball	High	High	Low	Low	Mod	Mod	Low	Low
Ice Hockey	High	High	Low	Low	Mod	High	Mod	Mod
Football	High	High	Mod	Mod	Mod	High	Mod	Mod
Baseball	High	High	Mod	Mod	Low	Low	Low	Low
Softball	High	High	Mod	Mod	Low	Low	Low	Low
Volleyball	High	High	Low	Low	Low	Low	Low	Low
Soccer	Mod	Low	Mod	Mod	Mod	High	Mod	High
Lacrosse	High	High	Mod	Mod	Mod	Mod	Mod	Mod
Rugby	High	Low	Mod	Mod	Mod	High	Mod	High

Availability of Fluid: High, the dynamics of the sport allow for multiple opportunities to consume fluid; Moderate (Mod), Fluid is only available during breaks in training, competition, or carried by the athlete; Low, Fluid is limited or not available due to time restrictions, rules or dynamics of the sport, and ability to carry. Environment: High, environmental conditions that are of great risk for hypohydration; Mod, the environment is variable ranging from cool to hot conditions that may pose risk for hypohydration; Low, the environmental conditions are not a threat to hypohydration. Intensity: High, exercise intensity in the sport is increased and likely to result in large sweat losses and hypohydration; Mod, exercise intensity in the sport varies from moderate to high and may result in large sweat losses and hypohydration; Low, exercise intensity in the sport is low and less likely to result in large sweat losses and hypohydration. Hypohydration Risk: High, the risk for hypohydration in the sport is high based on reported sweat losses, the availability of fluid, environmental conditions, and the intensity of exercise; Mod, the risk for hypohydration in the sport is moderate based on reported sweat losses, the availability of fluid, environmental conditions, and the intensity of exercise; Low, the risk for hypohydration in the sport is low based on reported sweat losses, the availability of fluid, environmental conditions, and the intensity of exercise. Note: These assessments are representative of typical situations encountered in these sports. Site-specific factors (Figure 1) may differ from those presented here.

**Table 2:** Individual Sport Factors That Influence Hypohydration.

Sport	Availability of Fluid		Environment		Intensity		Hypohydration Risk	
	Training	Competition	Training	Competition	Training	Competition	Training	Competition
Tennis	High	Mod	Mod	Mod	High	High	Mod	Mod
Wrestling	High	High	Mod	Mod	High	High	High	Low
Gymnastics	High	High	Low	Low	Mod	Low	Low	Low
Running (<1 h)	Low	High	Mod	Mod	High	High	Low	Low
Running (1–2 h)	Low	High	Mod	Mod	Mod	Mod	Mod	Mod
Running (>2 h)	Low	High	Mod	Mod	Low	Mod	Mod	Mod
Cycling (<1 h)	High	High	Mod	Mod	High	High	Low	Low
Cycling (>2 h)	Mod	Mod	Mod	Mod	Mod	Mod	Low	High
Swimming	High	High	Low	Low	High	High	Low	Low
Triathlon (<2 h)								
Swim	Low	Low	Low	Low	Mod	Mod	Low	Low
Bike	Mod	High	Mod	Mod	Mod	Mod	Low	Low
Run	Low	High	Mod	Mod	Mod	Mod	Low	Low
Triathlon (2–5 h)								
Swim	Low	Low	Low	Low	Mod	Mod	Low	Low
Bike	Mod	High	Mod	Mod	Mod	Mod	Low	Low
Run	Low	High	Mod	Mod	Mod	Mod	Low	Low
Triathlon (5–9 h)								
Swim	Low	Low	Low	Low	Mod	Mod	Low	Low
Bike	Mod	High	Mod	Mod	Mod	Mod	Mod	Mod
Run	Low	High	Mod	Mod	Mod	Mod	Mod	Mod
Triathlon (>9 h)								
Swim	Low	Low	Low	Low	Mod	Mod	Low	Low
Bike	Mod	High	Mod	Mod	Mod	Mod	Mod	Mod
Run	Low	High	Mod	Mod	Mod	Mod	Mod	Mod

Availability of Fluid: High, the dynamics of the sport allow for multiple opportunities to consume fluid; Moderate (Mod), Fluid is only available during breaks in training, competition, or carried by the athlete; Low, Fluid is limited or not available due to time restrictions, rules or dynamics of the sport, and ability to carry. Environment: High, environmental conditions that are of great risk for hypohydration; Mod, the environment is variable ranging from cool to hot conditions that may pose risk for hypohydration; Low, the environmental conditions are not a threat to hypohydration. Intensity: High, exercise intensity in the sport is increased and likely to result in large sweat losses and hypohydration; Mod, exercise intensity in the sport varies from moderate to high and may result in large sweat losses and hypohydration; Low, exercise intensity in the sport is low and less likely to result in large sweat losses and hypohydration. Hypohydration Risk: High, the risk for hypohydration in the sport is high based on reported sweat losses, the availability of fluid, environmental conditions, and the intensity of exercise; Mod, the risk for hypohydration in the sport is moderate based on reported sweat losses, the availability of fluid, environmental conditions, and the intensity of exercise; Low, the risk for hypohydration in the sport is low based on reported sweat losses, the availability of fluid, environmental conditions, and the intensity of exercise. Note: These assessments are representative of typical situations encountered in these sports. Site-specific factors (Figure 1) may differ from those presented here.

**Table 3:** Establishing a Hydration Plan.

Guiding Question	Steps to Correct	Implementation Example
Are athletes in a state of optimal hydration?	Assess hydration status	Have scales available before and after practice to assess fluid deficits Measure fluid needs via sweat rate
Is the exercise prolonged or intense?	Increase availability of palatable fluids	Have more breaks during longer practices or more intense exercise Allow longer duration breaks
Is the exercise being performed in environmental conditions that lead to greater fluid losses?	Establish breaks based upon environmental conditions	Modify practice schedules utilizing WBGT to establish work-to-rest ratios that allow for adequate fluid intake
Is fluid available throughout the entire duration of exercise?	Fluid is made readily available for athletes If fluid is restricted (e.g., running races, soccer matches etc.), maximize opportunities for rehydration	Provide free access to fluids during practice Ensure athletes utilize breaks to rehydrate when opportunities are limited
Are there individuals with intrinsic risk factors?	Identify individuals with high sweat rates or other limits to optimal hydration Identify individuals whose thirst drive is not matched to their fluid losses during exercise Counsel and monitor these athletes	Test sweat rates of individuals who have issues with hydration Develop individual hydration plans for high-risk athletes
Are there sport-specific factors that need to be considered?	Counsel athletes on health and performance risks of utilizing dehydration for weight loss	Assess hydration status alongside weight measurements to promote healthy weight management

WBGT: Wet-bulb globe temperature.
